# Early epidemiologic and genomic insights from Sierra Leone’s first mpox cases, 2025

**DOI:** 10.11604/pamj.2026.53.177.49795

**Published:** 2026-04-29

**Authors:** Eric Nzirakaindi Ikoona, Gebrekrstos Negash Gebru, Martin Faye, Bridget Magoba, Doris Harding, Mary Magdalene Sinnah, Mustapha Jalloh, Fatou Diène Thiaw, Aminata Tigiedankay Koroma, Serigne Fallou Mbacké Ngom, Joseph Sam Kanu, James Sylvester Squire, Landry Gerald Boussiengui, Ndongo Dia, Ousmane Faye, Boubacar Diallo, Abdourahmane Sow, Mohamed Alex Vandi, Sartie Kenneh, Austin Demby, Foday Sahr

**Affiliations:** 1 National Public Health Agency, 42A Main Motors Road, Wilberforce, Freetown, Sierra Leone,; 2 Sierra Leone Field Epidemiology Training Program, Freetown, Sierra Leone,; 3 Département de Virologie, Institut Pasteur de Dakar, Rue Dr Calmette, BP 220, Dakar, Sénégal,; 4 Ministry of Health, Youyi Building, Brookfields, Freetown, Sierra Leone

**Keywords:** Mpox, outbreak investigation, rapid response, genomic surveillance, epidemiology, Sierra Leone, West Africa

## Abstract

**Introduction:**

early characterization of outbreak cases supports rapid decisions. We conducted real-time analysis during the initial outbreak phase in mid-March 2025 of Sierra Leone's first 44 laboratory-confirmed Mpox cases (10^th^ January to 5^th^ March 2025) to generate epidemiologic and genomic intelligence for response.

**Methods:**

we summarized surveillance data and sequenced 18 early cases with Oxford Nanopore. Firth-penalized logistic regression was employed to explore risk factors for severe disease.

**Results:**

median age was 27 years (interquartile range 22 to 35); 68.2% were male. Most cases reported no recent international travel (95.5%). All cases presented with rash; fever occurred in 72.7%. Six cases (13.6%) met severe criteria; no deaths occurred (0 of 44; 95% confidence interval 0 to 8.0). Household secondary attack rate was 7.3% during the study window and 8.2% after completion of follow-up. Sequencing identified two co-circulating sub-lineages consistent with A.2.2 and B.1.6 and a strong APOBEC3 pattern (142 of 212 guanine-to-adenine in thymine-cytosine versus 70 of 212 cytosine-to-thymine in guanine-adenine; exact binomial p approximately 8.6x10^-7^; X^2^= 24.5, df = 1, p≈ 7.6x10^-7^). Root-to-tip analysis showed weak temporal signal (R^2^=0.31; date randomization p= 0.18), so we did not interpret clock estimates.

**Conclusion:**

real-time analysis during the initial outbreak phase showed community transmission, quantified household spread, documented two sub-lineages with an APOBEC3 signature, and generated severity hypotheses that immediately informed surveillance and vaccine prioritization. Findings require validation in larger cohorts.

## Introduction

Mpox virus has evolved from a sporadic zoonotic disease primarily affecting Central and West Africa to one capable of sustained human-to-human transmission since the emergence of Clade IIb in 2022 [1-3]. The 2022 to 2024 global outbreak raised concerns about potential endemic establishment beyond historically affected regions and underscored the need for enhanced genomic surveillance to track viral evolution [4-6]. Transmission patterns differ markedly across geographic contexts. Outbreaks in Europe and North America demonstrated predominant spread through sexual contact networks, particularly among men who have sex with men, with genital lesions occurring in 36% to 73% of cases [7-9]. In contrast, West and Central African outbreaks primarily exhibit household and community-based transmission [10-12]. Disease severity varies across population groups, with older adults, individuals with comorbidities, and immunocompromised patients facing higher risks [13-15]. Despite Africa bearing the highest historical burden of mpox, the continent remains significantly underrepresented in genomic sequencing efforts, hindering real-time monitoring of viral mutations and transmission dynamics [16-18].

Sierra Leone confirmed its first Clade IIb Mpox case on 10^th^ January 2025, prompting the declaration of a public health emergency on 13^th^ January 2025. Previous mpox detection in Sierra Leone occurred sporadically in 1970, 2014, and 2017, with long intervals of undetected circulation [19-21]. Unlike these historical sporadic cases linked to zoonotic exposure, early epidemiological assessment suggested sustained human-to-human transmission requiring urgent characterization. The absence of routine genomic surveillance in Sierra Leone and neighboring countries had limited efforts to track viral introductions and detect emerging mutations [22]. Limited diagnostic capacity and delayed case detection created a risk of prolonged transmission chains.

The first weeks of an outbreak represent a critical window to establish surveillance systems, define transmission patterns, and characterize clinical severity to guide response strategies. In March 2025, during the initial phase of Sierra Leone's outbreak, we conducted a rapid analysis of the first 44 laboratory-confirmed mpox cases (10^th^ January-5^th^ March 2025). Despite the small sample and inherent uncertainties, this early characterization provided actionable epidemiological and genomic intelligence that directly informed surveillance priorities, vaccine allocation, and genomic partnerships. Findings were shared in real time with response leadership and translated into immediate public health action. Here, we present the full analysis and its impact as the outbreak evolved.

## Methods

### Study design, period, and setting

**Figure 1:**
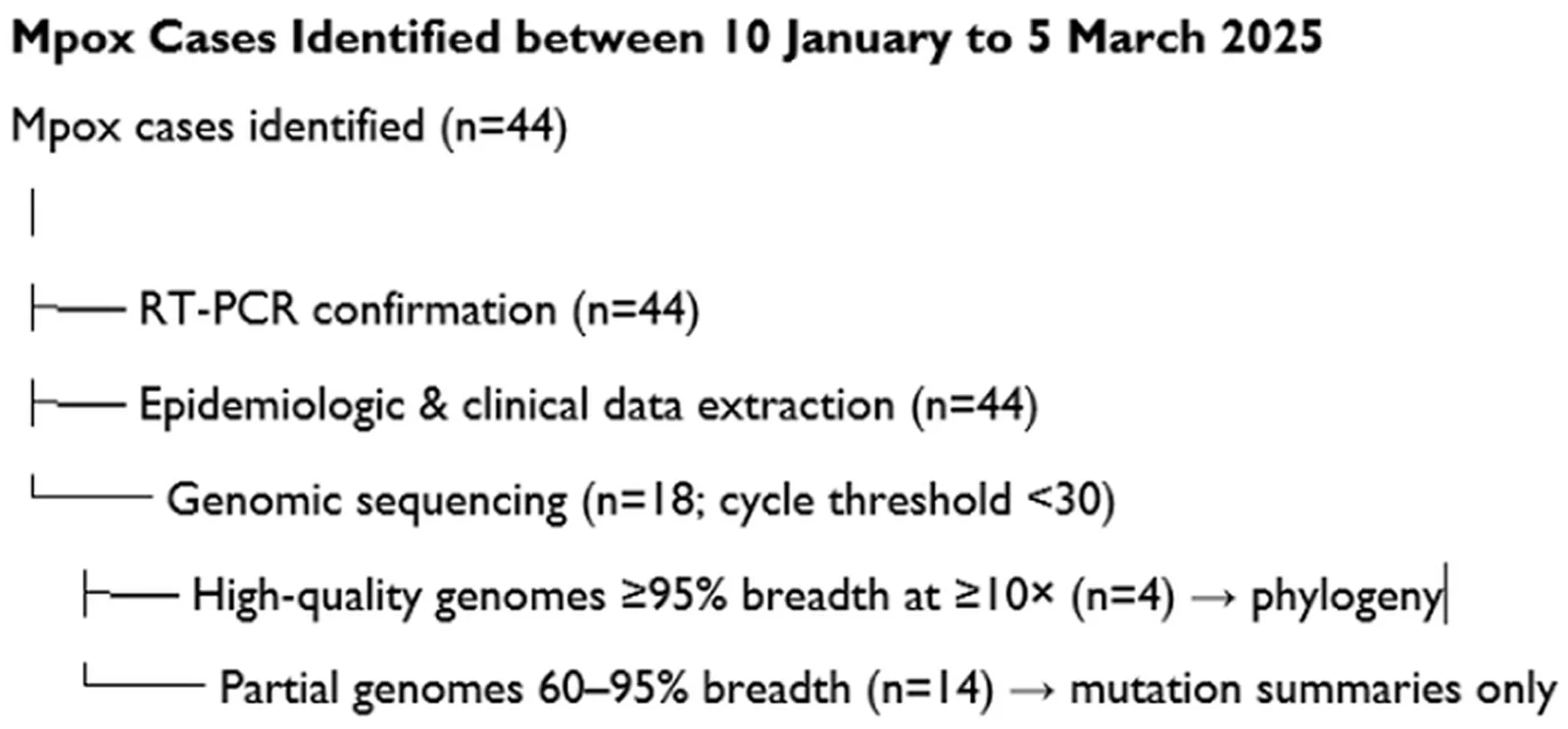
diagnostic and genomic workflow (January-March 2025)

We conducted real-time observational analysis during the initial outbreak phase of all 44 laboratory-confirmed mpox cases identified in Sierra Leone between 10^th^ January and 5^th^ March 2025. Analysis was performed in mid-March 2025 (6^th^ to 17^th^ March), while the outbreak was actively evolving, to provide timely intelligence for response strategy development. Data were extracted from the District Health Information System 2, the country's integrated surveillance platform, by authorized National Public Health Agency personnel following data protection protocols. The investigation included all districts reporting confirmed cases during this period. Figure 1 summarizes the diagnostic and genomic sequencing workflow: all 44 identified cases underwent RT-PCR confirmation and epidemiologic data extraction, with 18 cases (those with cycle threshold values below 30) selected for genomic sequencing, yielding 4 high-quality genomes suitable for phylogenetic analysis and 14 partial genomes used for mutation analysis only (Figure 1).

### Case definitions and disease classification

Cases were classified according to National Public Health Agency criteria aligned with World Health Organization and United States Centers for Disease Control and Prevention guidelines [23,24]. A suspected case was defined as an individual presenting with unexplained acute skin rash, mucosal lesions, or lymphadenopathy within 21 days, accompanied by at least one systemic symptom including fever exceeding 38.5°C, headache, myalgia, back pain, or profound weakness. A probable case met suspected case criteria with an epidemiological link to a confirmed or probable case within 21 days. A confirmed case had laboratory-detected Monkeypox virus DNA through real-time polymerase chain reaction.

Severe disease was classified according to World Health Organization criteria as cases presenting with severe pain requiring parenteral analgesia, lower respiratory tract involvement confirmed by clinical examination or radiography, ocular complications including keratitis or corneal ulceration, neurologic impairment including encephalitis or seizures, cardiac dysfunction, clinical dehydration requiring intravenous fluid replacement, progressive or extensive lesions involving greater than 50% of body surface area, or severe proctitis, urethritis, or balanitis requiring specialized care [23]. Disease severity was assessed by experienced clinicians at treatment facilities, with review by the outbreak investigation team. Cases were classified at the time of maximum severity during the clinical course.

### Data collection and contact tracing

Demographic and clinical data were collected through standardized case report forms completed by clinical staff at patient presentation. Human immunodeficiency virus status was documented only when patients had prior known diagnosis or presented clinical information; systematic testing was not performed as part of routine mpox case investigation. Exposure history focused on household and close contact identification for contact tracing purposes. Smallpox vaccination history was not systematically ascertained; given that routine smallpox vaccination ceased globally by 1980, individuals over 45 years of age might have childhood vaccination [25]. Comorbidity ascertainment relied on patient self-report, review of available medical records, and clinical assessment at presentation.

Contact tracing was initiated for all confirmed cases following national protocols. Contacts were defined as individuals who had face-to-face contact with a confirmed case within 1 meter for three hours or more, direct physical contact with lesions or body fluids, or shared household or sleeping arrangements during the infectious period extending from symptom onset until all lesions had crusted and separated [23]. Contacts were monitored daily for 21 days from last exposure. Secondary attack rates were calculated as the proportion of contacts who developed confirmed mpox disease during follow-up.

### Laboratory methods and genomic sequencing

Laboratory confirmation was performed at three designated facilities through real-time polymerase chain reaction using the Abbott m2000 RealTime System. These facilities included the Central Public Health Reference Laboratory, the Kenema Viral Haemorrhagic Fever Laboratory, and the Jui P3 Laboratory. Testing utilized World Health Organization-recommended primers targeting G2R_G and G2R_WA genes [23]. Specimens were transported in viral transport media under cold chain conditions. Cycle threshold values were recorded for all positive samples.

Whole-genome sequencing was performed on 18 of the 44 confirmed cases, representing the first consecutive confirmed cases where viable lesion swab or lesion crust specimens with cycle threshold values below 30 were available. Specimens were shipped to *Institut Pasteur*, Dakar, under appropriate biosafety conditions maintaining cold chain integrity. Viral DNA was extracted using the QIAamp DNA Mini Kit (Qiagen, Germany) following manufacturer protocols. Amplification utilized an amplicon-based method with the Oxford Nanopore rapid barcoding kit according to manufacturer recommendations [26]. Barcoded samples were pooled in equal volume and purified using magnetic beads. The purified libraries were adapter-ligated, loaded on a primed R10.4.1 flow cell, and subjected to whole genome sequencing on a GridION instrument (Oxford Nanopore Technologies). Raw sequencing reads were quality-filtered, adapter-trimmed, and mapped to the Monkeypox virus reference genome (GenBank accession NC_063383) using minimap2, and consensus sequences were generated using the Chan Zuckerberg ID web platform.

Sequences achieving at least 95% genome coverage at a minimum of 10-fold depth were classified as high-quality and used for phylogenetic reconstruction. Sequences with coverage between 60% and 95% were retained for mutation analysis but excluded from phylogenetic inference.

### Phylogenetic and evolutionary analysis

Phylogenetic relationships were inferred using the maximum likelihood estimation method implemented in IQ-TREE v2.0 with 1,000 bootstrap replications. The GTR+F+I+G4 nucleotide substitution model was selected based on model comparison criteria. Trees were visualized using FigTree v1.4.4. Consensus sequences and Clade IIb reference genomes from GenBank were aligned using Squirrel v1.0.11.

### Temporal signal and exploratory clock analysis

We assessed temporal signal in TempEst v1.5.3 through root-to-tip regression (R^2^ = 0.31) and performed date-randomization testing with 100 permutations (not different from random, p = 0.18). We then ran an exploratory Bayesian analysis in BEAST v1.10.4 using a relaxed log-normal clock, GTR+F+I+G4 substitution model, and constant coalescent tree prior. Markov Chain Monte Carlo chains ran for 100 million generations, sampling every 10,000 steps; convergence was assessed in Tracer v1.7. Because temporal signal was weak and the sampling window spanned only eight weeks, all clock outputs were treated as exploratory. The median time to Most Recent Common Ancestor (tMRCA) with 95% Highest Posterior Density (HPD) intervals was reported for context only, without rate- or timing-based inference. Detailed BEAST settings, priors, and diagnostics appear in Supplementary File I.

### Mutation and lineage analysis

**Figure 2:**
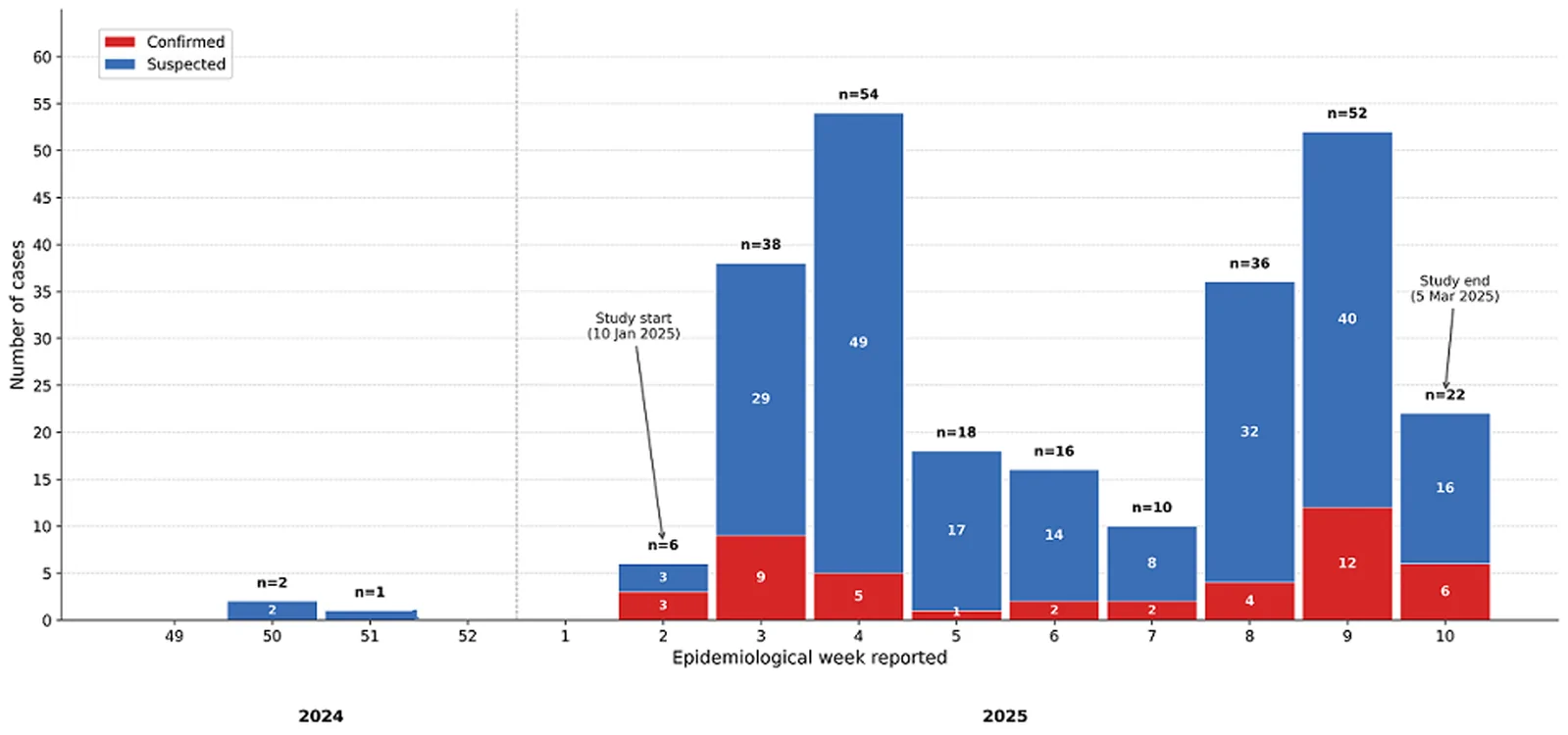
weekly suspected and confirmed mpox cases, Sierra Leone, epidemiological week 49 (2024) - week 10 (2025)

Mutation analysis compared each genome to the Monkeypox virus reference sequence (GenBank accession NC_063383). Substitutions were classified by type and dinucleotide context to identify APOBEC3-associated editing signatures. Functional impacts of non-synonymous variants were predicted using PROVEAN v1.1.3 with the default deleterious threshold of -2.5. Lineage assignment employed Nextclade CLI v3.2.2 using the mpox database build 2025-03-15, consistent with the Pathoplexus repository version corresponding to accession numbers PP_0049Q1L.1, PP_0049Q0N.1, PP_0049PZQ.1, and PP_0049PYS.1. Lineage designations (A.2.2, B.1.6) follow that database's nomenclature. Mutation distributions are summarized in Figure 2.

### Statistical analysis

Statistical analyses were conducted using R version 4.1.3. We summarized categorical variables using frequencies and percentages with 95% confidence intervals calculated using the Clopper-Pearson exact method. Age was described using median and interquartile range given non-normal distribution. We examined associations between predictor variables and severe disease using Firth-penalized logistic regression, which reduces small-sample bias compared to standard maximum likelihood estimation [27,28]. Predictor variables included age group (35 years or older versus less than 35 years), presence of any documented comorbidity, lesion distribution (generalized versus localized), sex, and recent travel history. We conducted bivariate Firth-penalized logistic regression for each predictor, then constructed a parsimonious multivariable model including age group and presence of comorbidities based on strongest bivariate associations and biological plausibility. Profile-likelihood confidence intervals were calculated for all estimates. Given only six severe events, all analyses were explicitly framed as hypothesis-generating rather than definitive. We compared APOBEC-context counts against a 50:50 null using a chi-square goodness-of-fit test (1 degree of freedom) and reported the exact two-sided binomial p-value.

### Ethical approval

This study analyzed de-identified surveillance data collected through routine public-health response activities during a declared emergency. Permission was obtained from National Public Health Agency leadership. Individual patient consent was not required under national guidelines for emergency outbreak investigations. The study followed ethical principles for public-health research consistent with World Health Organization recommendations for outbreak response. All personally identifiable information was removed from analytical datasets, and geographic information was retained only at the district level. Genomic sequences submitted to public repositories (Pathoplexus) include only sample collection dates, districts, and technical metadata without any patient identifiers. We followed the STROBE guideline for observational studies.

### Data availability

De-identified surveillance data analyzed in this study are available from the corresponding author (ENI) upon reasonable request and with appropriate data-sharing agreements to ensure confidentiality. Genomic sequences have been submitted to Pathoplexus webform under the following accession numbers: PP_0049Q1L.1, PP_0049Q0N.1, PP_0049PZQ.1 and PP_0049PYS.1. Analysis code and BEAST parameter files will be archived on a public repository (Zenodo or GitHub) upon acceptance.

## Results

### Demographic and epidemiological characteristics

**Figure 3:**
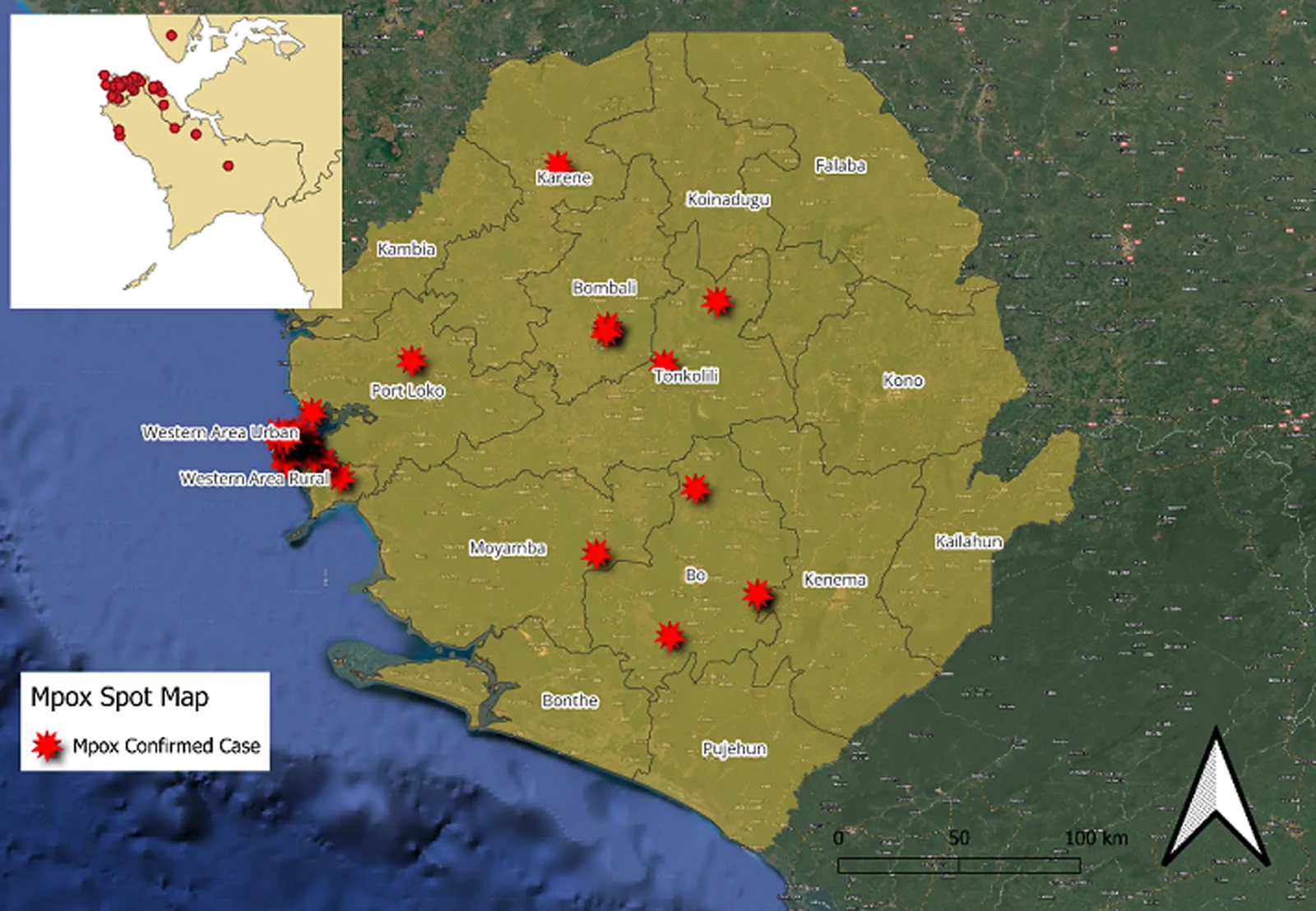
geographic distribution of mpox cases by district, Sierra Leone, 10 January to 5 March 2025

Between 10^th^ January and 5^th^ March 2025, 44 laboratory-confirmed mpox cases were reported across eight districts in Sierra Leone (Table 1). The median age was 27 years (interquartile range 22 to 35 years), with 13 cases (29.5%, 95% confidence interval 15.2% to 47.5%) aged 35 years or older (Table 1). Males comprised 68.2% of cases (30 of 44, 95% confidence interval 51.3% to 82.5%). Most cases (42 of 44, or 95.5%, 95% confidence interval 85.1% to 98.8%) reported no travel outside Sierra Leone in the 21 days before symptom onset (Table 1). Two cases reported recent travel to neighbouring countries. One confirmed case occurred in a healthcare worker. One case occurred in a pregnant woman in her second trimester who subsequently delivered a healthy infant. Cases were concentrated in Western Area Urban (22 cases, 50.0%) and Western Area Rural (11 cases, 25.0%). Additional cases were reported in Bombali (4 cases), Tonkolili (3 cases), Bo (2 cases), Port Loko (1 case), and Kenema (1 case) (Figure 3).

**Table 1:** demographic, clinical, and epidemiological characteristics of the first 44 mpox cases, Sierra Leone, 10^th^ January to 5^th^ March 2025

Characteristic	n (%) or median (IQR)	95% CI
**Demographics**		
Total cases	44 (100)	-
Age, years	27 (22-35)	-
Age ≥35 years	13 (29.5)	15.2-47.5
Male sex	30 (68.2)	51.3-82.5
Healthcare worker	1 (2.3)	0.1-12.9
Pregnant	1 (2.3)	0.1-12.9
**Geographic distribution**		
Western Area Urban	22 (50.0)	34.6-65.4
Western Area Rural	11 (25.0)	13.2-40.5
Bombali	4 (9.1)	2.8-22.5
Tonkolili	3 (6.8)	1.8-19.7
Bo	2 (4.5)	0.8-16.5
Port Loko	1 (2.3)	0.1-12.9
Kenema	1 (2.3)	0.1-12.9
**Epidemiological factors**		
No recent travel history	42 (95.5)	85.1-98.8
Travel to neighboring country	2 (4.5)	1.2-14.9
**Clinical presentation**		
Rash/lesions	44 (100)	91.9-100
Fever (>38.5°C)	32 (72.7)	56.8-85.4
Headache	28 (63.6)	47.2-77.9
Myalgia	19 (43.2)	28.4-59.1
Lymphadenopathy	12 (27.3)	15.3-42.9
Genital/perianal lesions	5 (11.4)	4.3-24.7
**Disease severity**		
Severe disease	6 (13.6)	6.4-26.5
Hospitalized	6 (13.6)	6.4-26.5
Deaths	0 (0)	0-8.0
**Comorbidities**		
Any documented comorbidity	8 (18.2)	9.2-32.7
Diabetes mellitus	3 (6.8)	1.8-19.7
Hypertension	3 (6.8)	1.8-19.7
Chronic kidney disease	1 (2.3)	0.1-12.9
Known HIV infection	1 (2.3)	0.1-12.9
**Lesion distribution**		
Generalized (≥3 regions)	19 (43.2)	28.4-59.1
Localized (<3 regions)	25 (56.8)	40.9-71.6
**Time intervals**		
Symptom onset to presentation, days	5 (3-8)	-
Symptom onset to confirmation, days	8 (6-11)	-
**Contact tracing**		
Total contacts identified	413	-
Contacts per case	9 (6-14)	-
Secondary cases during study window	30 (7.3)	5.0-10.3
Cumulative secondary cases after complete follow-up	34 (8.2)	5.8-11.3
Contacts completed follow-up	366 (88.6)	85.1-91.5
Contacts under monitoring at study end	40 (9.7)	7.0-13.0
Contacts lost to follow-up	7 (1.7)	0.7-3.5

**Abbreviations:** CI, confidence interval; HIV, human immunodeficiency virus; IQR, interquartile range

### Contact tracing outcomes

Contact tracing identified 413 contacts across all confirmed cases (Table 1). At the study completion date (5^th^ March 2025), 30 contacts had developed laboratory-confirmed mpox, yielding a secondary attack rate during the study window of 7.3% (95% confidence interval 5.0% to 10.3%). At this date, 366 contacts (88.6%) had completed the full 21-day follow-up period without developing symptoms, 40 contacts (9.7%) remained under active monitoring, having not yet completed 21 days of follow-up, and 7 contacts (1.7%) were lost to follow-up after initial assessment. Following study completion, the 40 contacts still under monitoring were followed to conclusion. Of these, 36 completed follow-up without developing symptoms while 4 developed confirmed mpox. Including these post-study cases, the cumulative secondary attack rate was 8.2% (34 of 413 contacts, 95% confidence interval 5.8% to 11.3%).

### Clinical presentation and disease severity

All 44 cases presented with cutaneous lesions affecting primarily the face, trunk, and extremities. Fever exceeding 38.5°C occurred in 32 cases (72.7%, 95% confidence interval 56.8% to 85.4%). Lymphadenopathy, predominantly cervical and inguinal, was documented in 12 cases (27.3%, 95% confidence interval 15.3% to 42.9%). Headache was reported in 28 cases (63.6%, 95% confidence interval 47.2% to 77.9%). Myalgia occurred in 19 cases (43.2%, 95% confidence interval 28.4% to 59.1%). Five cases (11.4%, 95% confidence interval 4.3% to 24.7%) presented with genital or perianal lesions. Eight cases (18.2%, 95% confidence interval 9.2% to 32.7%) had documented comorbidities including diabetes mellitus (3 cases), hypertension (3 cases), chronic kidney disease (1 case), and known human immunodeficiency virus infection (1 case). Nineteen cases (43.2%, 95% confidence interval 28.4% to 59.1%) presented with generalized lesion distribution involving three or more non-contiguous body regions.

Six cases (13.6%, 95% confidence interval 6.4% to 26.5%) developed severe disease requiring hospitalization or advanced clinical management. Three developed lower respiratory tract involvement with confirmed pneumonitis on chest radiography. Two cases developed severe secondary bacterial skin infections requiring parenteral antibiotics. One case developed ocular complications with keratitis requiring ophthalmologic consultation. Three severe cases required parenteral analgesia for severe pain management. All severe cases required hospitalization with a median hospital stay of 16 days (range 8 to 21 days). One severe case developed bacteremia requiring intensive management but ultimately recovered. No deaths occurred among the first 44 cases.

The median time from symptom onset to healthcare presentation was 5 days (interquartile range 3 to 8 days). The median time from symptom onset to laboratory confirmation was 8 days (interquartile range 6 to 11 days). For severe cases specifically, the median time to developing severe complications was 7 days after symptom onset (range 4 to 12 days).

### Temporal distribution

**Figure 4:**
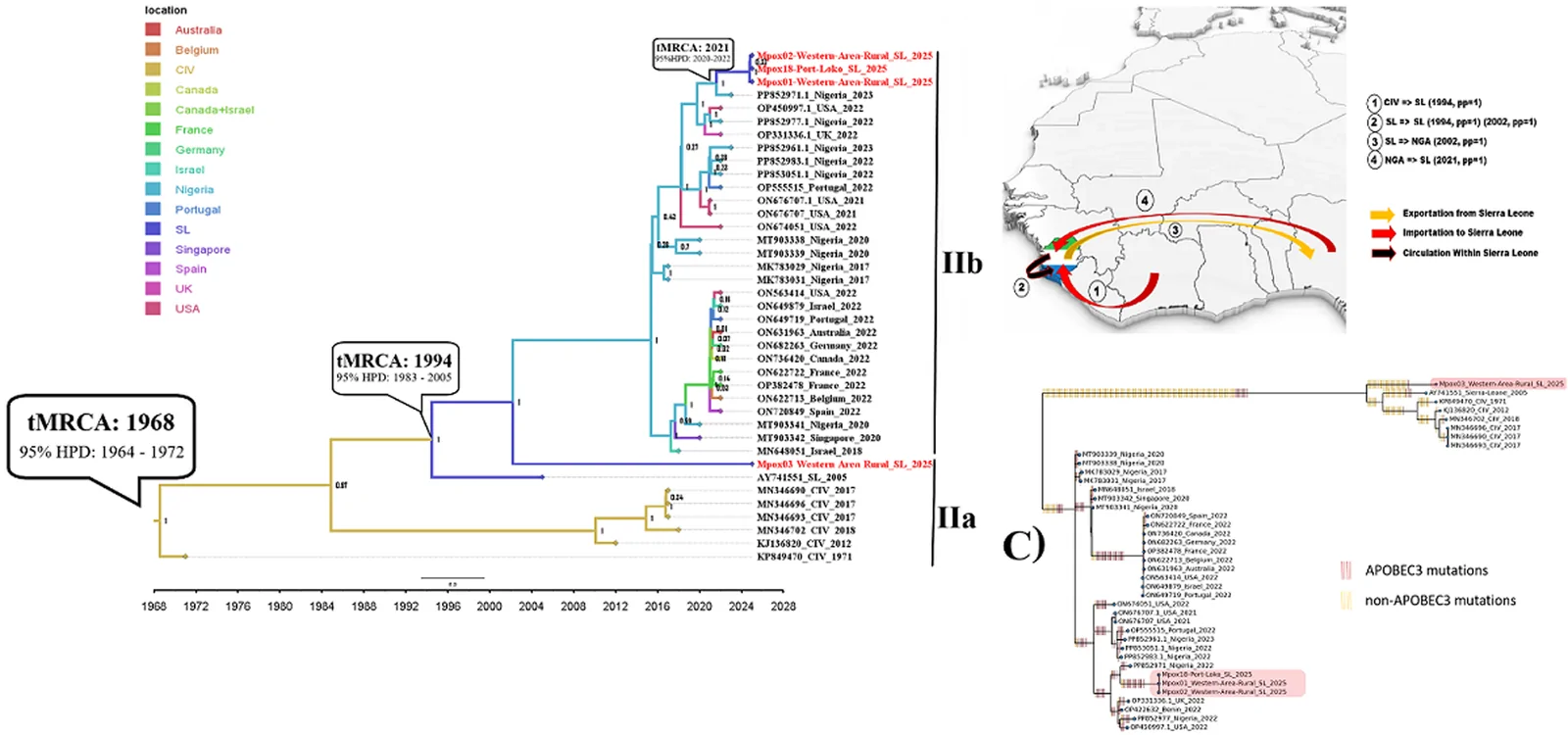
genomic characterization of early mpox cases in Sierra Leone

The epidemic curve demonstrated an initial peak in case detection during epidemiological week 4 (22^th^ to 28^th^ January 2025) with 14 cases reported. This was followed by a gradual decline over the subsequent two weeks, then a secondary rise during epidemiological week 7 (12^th^ to 18^th^ February 2025) with 9 cases reported (Figure 4).

### Genomic characterization

Of the 18 samples submitted for sequencing, 4 achieved high-quality coverage exceeding 95% of the reference genome at a minimum 10-fold depth (mean depth range 387 to 612x, mean 498x). Fourteen samples achieved partial coverage between 60% and 85% (mean depth range 45 to 156x). Cycle threshold values for samples achieving high-quality sequences ranged from 18.2 to 27.4 (median 23.1), while partially sequenced samples had cycle threshold values ranging from 24.8 to 29.8 (median 27.9).

All sequenced samples were consistent with Clade IIb based on phylogenetic placement and characteristic mutations. Analysis of the four high-quality sequences revealed two genetically distinct groups designated A.2.2 and B.1.6 according to Nextclade nomenclature. The A.2.2 sub-lineage, identified in three sequences (PP_0049Q1L.1, PP_0049PZQ.1 and PP_0049PYS.1), showed high nucleotide identity (99.2%) to recently sequenced strains from Nigeria. The B.1.6 sub-lineage, identified in one sequence (PP_0049Q0N.1), showed 98.7% nucleotide identity to strains from Côte d'Ivoire. Bootstrap support values for the phylogenetic split between these two groups were 98% and 95%, respectively. The temporal distribution of cases infected with each lineage showed overlapping circulation throughout the study period (Figure 2).

Mutation analysis across 18 sequenced samples found 212 changes relative to NC_063383. Of these, 142 (67.0%) were guanine-to-adenine transitions in thymine-cytosine dinucleotide contexts and 70 (33.0%) were cytosine-to-thymine transitions in guanine-adenine dinucleotide contexts (X^2^= 24.5, df = 1, p ≈ 7.6x10^-7^). An exact two-sided binomial test gave p approximately 8.6x10^-7^. Among mutations detected, 119 were synonymous and 93 were non-synonymous.

Clock signal and exploratory tMRCA. Root-to-tip analysis showed limited temporal signal (R^2^=0.31), and date-randomization testing showed no difference from random (p=0.18). We still ran an exploratory BEAST analysis that placed the tMRCA near 1968 (95% Highest Posterior Density [HPD] 1964-1972) and suggested windows of historical introductions in the 1990s, early 2000s, and around 2021. These dates provide historical context only and do not support rate- or clock-based inference in this dataset.

### Exploratory risk factor analysis for severe disease

Forty-four cases contributed to exploratory risk factor analysis for severe disease; six met the severe endpoint (Table 2). In bivariate Firth-penalized logistic regression, presence of any documented comorbidity showed association with severe disease (odds ratio 43.8, 95% confidence interval 4.7 to 412.3, p=0.001). Age 35 years or older showed a positive association (odds ratio 6.3, 95% confidence interval 0.9 to 44.2, p=0.061). Generalized lesion distribution showed a positive association (odds ratio 3.0, 95% confidence interval 0.5 to 19.8, p=0.25). Male sex and recent travel history showed no evidence of association.

**Table 2:** exploratory analysis of factors associated with severe disease using firth-penalized logistic regression (n= 44 cases, 6 severe)

Variable	Severe n/N (%)	Bivariate OR (95% CI)	p-value	Multivariable aOR (95% CI)	p-value
**Age group**					
<35 years	2/31 (6.5)	1.0 (Reference)	-	1.0 (Reference)	-
≥35 years	4/13 (30.8)	6.3 (0.9-44.2)	0.061	4.5 (0.6-34.9)	0.14
**Comorbidities**					
None documented	1/36 (2.8)	1.0 (Reference)	-	1.0 (Reference)	-
Any documented	5/8 (62.5)	43.8(4.7-412.3)	0.001	31.2 (3.2-304.7)	0.003
**Lesion distribution**					
Localized	2/25 (8.0)	1.0 (Reference)	-	-	-
Generalized	4/19 (21.1)	3.0 (0.5-19.8)	0.25	-	-
**Sex**					
Female	1/14 (7.1)	1.0 (Reference)	-	-	-
Male	5/30 (16.7)	2.6 (0.3-25.4)	0.41	-	-
**Travel history**					
No recent travel	6/42 (14.3)	1.0 (Reference)	-	-	-
Recent travel	0/2 (0)	NC	0.99	-	-

Abbreviations: aOR, adjusted odds ratio; CI, confidence interval; NC, not calculable; OR, odds ratio. Note: this exploratory analysis with only 6 severe cases generates hypotheses for validation in larger cohorts. Wide confidence intervals reflect inherent statistical uncertainty with small event numbers. These findings informed vaccine prioritization discussions in March 2025 alongside other evidence sources but should not be used for clinical decision-making without validation in substantially larger studies. The multivariable model includes age group and presence of comorbidities based on strongest bivariate associations and biological plausibility.

In the parsimonious multivariable model including age group and presence of comorbidities, documented comorbidity remained associated with severe disease (adjusted odds ratio 31.2, 95% confidence interval 3.2 to 304.7, p=0.003). Age 35 years or older showed a non-significant trend after adjustment (adjusted odds ratio 4.5, 95% confidence interval 0.6 to 34.9, p=0.14). The extremely wide confidence intervals reflect the fundamental limitation of analyzing only six severe events.

## Discussion

### Rapid outbreak characterization to inform real-time response

Analysis of the first 44 mpox cases in Sierra Leone provided early evidence that shaped surveillance, clinical care, and vaccine strategies. Despite the small sample, findings remained consistent as the outbreak expanded to 5,426 cases by October 2025 [29], affirming the utility of rapid characterization in early response. Community-based transmission predominated, with 95.5% of cases lacking travel history and concentration in urban areas. This prompted a strategic pivot in late March 2025 from border-focused surveillance to localized case finding and contact tracing. The household secondary attack rate of 7.3% to 8.2% quantified transmission intensity in close-contact settings. Low prevalence of genital lesions (11.4%) contrasted sharply with 36% to 73% in European outbreaks [7-9], supporting tailored risk communication for Sierra Leone.

Genomic sequencing revealed two distinct sub-lineages (A.2.2 and B.1.6), likely representing separate introductions from Nigeria and Côte d’Ivoire, reinforcing the need for genomic surveillance. Exploratory logistic regression, though based on only six severe cases, generated plausible risk hypotheses: individuals 35 years or older and those with comorbidities had elevated odds of severe illness. These findings informed vaccine prioritization when limited doses became available in late March 2025. Despite wide confidence intervals, patterns identified early proved epidemiologically durable as the outbreak expanded [29].

### Translation of early findings to response strategy

Clinical teams adapted protocols based on observed complications, including pneumonitis, bacterial infections, and ocular involvement among severe cases. Moderate severity rates without fatalities supported existing supportive care while emphasizing early complication management. Demographic patterns-predominantly young male adults-resembled the 2022 to 2023 multi-country outbreaks [7-9], though transmission contexts differed. These insights informed risk communication strategies.

Risk factor data informed the national vaccination framework launched 27^th^ March 2025. Despite statistical limitations, our analysis supported prioritizing adults 35 years or older, people with chronic conditions, and individuals with generalized rashes, consistent with evidence from other settings [13-15]. By October 2025, 170,141 vaccine doses had been administered, though coverage remained below 2.5% due to supply limitations [29]. The outbreak's subsequent evolution validated early observations. By October 2025, case fatality rate (1.0%) and age distribution remained consistent with initial findings [29], with geographic concentration persisting in Western Area Urban. These continuities reinforced the value of early data in shaping scalable response systems.

### Regional context and transmission dynamics

Sierra Leone's outbreak differed from prior mpox patterns observed in West Africa. Nigeria's 2017 to 2019 outbreaks were characterized by zoonotic spillover events with limited human-to-human transmission, typically 3 to 4 cases per chain [30]. In contrast, Sierra Leone showed sustained community transmission, longer chains (7 to 8 cases), and a higher proportion of urban cases (67% versus 42%). Ghana's 2022 outbreak was similarly zoonotic, with a lower secondary attack rate (3.8%) compared to the 7.3% to 8.2% observed here [31]. Phylogenetic data confirmed at least two independent viral introductions into Sierra Leone, with sustained local transmission, contrasting with the primarily travel-associated cases seen in Côte d'Ivoire [32]. Observed patterns aligned more closely with Central and West African outbreaks, where household and community-based transmission predominate [10-12].

The household secondary attack rate in Sierra Leone falls between historical smallpox (37% to 88%) [25] and influenza (5% to 15%) [33], consistent with other African mpox settings [10,11]. The 13.6% severe disease rate exceeded that of recent outbreaks in Europe and North America (1% to 3%) [7] but was lower than reported rates from Central Africa (20% to 30%) [10,11]. These differences likely reflect variation in healthcare access, population age structure, and immunological status. The observed comorbidity associations are consistent with studies linking severity to chronic disease and immunocompromised status [13-15].

This outbreak occurred alongside Clade I mpox epidemics in the Democratic Republic of Congo, Uganda, and Burundi, which had different transmission patterns and clinical severity [34,35]. While comparisons are limited by lineage differences, the regional context reinforces the need for tailored outbreak response frameworks. International partners including WHO, CDC, Africa CDC, and others provided essential support for diagnostics, contact tracing, and vaccine access. However, reliance on donor-provided vaccines restricted scale-up [29]. By October 2025, Sierra Leone had traced 22,396 contacts, compared to just 413 during our initial study period [29]. Declining contact listings per case likely reflected operational fatigue rather than reduced transmission. The geographic spread from 8 to all 16 districts illustrated the challenge of containing community transmission in low-resource settings [29]. Despite extensive effort, the outbreak's expansion emphasized the need for scalable, decentralized, and sustained response infrastructure.

### Genomic surveillance: from early detection to sustained monitoring

Detection of co-circulating A.2.2 and B.1.6 sub-lineages linked to Nigeria and Côte d'Ivoire [36] reinforced the need for sustained genomic surveillance to monitor and track variant emergence and cross-border transmission. The strong APOBEC3 mutational signature (67% guanine-to-adenine in thymine-cytosine contexts) confirmed host-driven evolution during human-to-human transmission [16-18,37,38], a hallmark of Clade IIb outbreaks. Through regional partnerships, Sierra Leone scaled genomic sequencing capacity to exceed the 10% threshold recommended for real-time monitoring [29]. Functional analysis identified non-synonymous mutations in immunogenic loci F13L and A29L, though phenotypic impacts remain unconfirmed [39,40]. Exploratory timing signals. Root-to-tip regression revealed insufficient temporal signal (R^2^= 0.31, p= 0.18) for reliable molecular clock analysis [41]. The exploratory clock output suggests deep ancestry of the Sierra Leone genomes around 1968 (95% HPD 1964-1972) with possible introduction windows in the 1990s, early 2000s, and near 2021 [42]. The temporal signal is weak, and sampling spans only eight weeks, so we interpret these dates as hypotheses rather than estimates for decision-making. Denser longitudinal sampling will test these hypotheses.

### Strengths and limitations of rapid outbreak characterization

This analysis provides one of the earliest integrated epidemiological and genomic profiles of Sierra Leone's mpox outbreak. However, it is limited by small sample size (n= 44), low event counts (six severe cases), and partial genomic coverage (four high-quality sequences). This constrained statistical power and phylogenetic resolution. Potential biases include overrepresentation of severe cases, underascertainment of comorbidities, and lack of systematic HIV or behavioral data. Documented comorbidity prevalence (18.2%) likely underestimates the true rate, given population-level estimates of hypertension and diabetes in West Africa [43,44]. Sample selection for sequencing favored low cycle threshold values, introducing bias.

The genomic analysis has important limitations. Root-to-tip regression revealed weak temporal signal (R^2^= 0.31, p= 0.18), substantially limiting molecular clock reliability. While exploratory Bayesian analysis estimated tMRCA at approximately 1968 with inferred introductions in 1994, 2002, and 2021, these estimates require cautious interpretation. Bayesian methods can produce point estimates even with inadequate temporal signal, but such estimates may be unreliable with misleadingly narrow confidence intervals. These findings are hypothesis-generating, suggesting possible long-term undetected circulation or repeated introductions, but require validation through longitudinal sampling spanning multiple years with substantially larger sample sizes.

Despite these limitations, the study met its objective: to inform early response with timely data. Community-based spread warranted localized surveillance. Lineage diversity justified sustained sequencing. Preliminary severity signals supported targeted vaccine allocation. These frameworks scaled nationally and proved durable as the outbreak evolved. The enduring value of early outbreak analysis lies in its ability to shape strategy under uncertainty. As shown here, rapid epidemiologic and genomic insights – even from limited data – can guide effective interventions when timing is critical.

### Research priorities emerging from early characterization

Future work should validate the association between severe disease, comorbidity, and older age in multicenter cohorts with systematic human immunodeficiency virus testing, CD4 T cell enumeration, and standardized severity assessment [13-15]. Studies should explain household transmission heterogeneity by measuring household size, isolation timing, and infection-prevention practices [10-12,33,45]. Population-based serosurveys should estimate total infection burden, detect asymptomatic infection, and define age-specific immunity patterns [2,6]. Studies on transmission dynamics should incorporate sexual network analysis and superspreading events [46,47]. Observational cohorts should characterize the full clinical spectrum including atypical presentations [48]. Sustained genomic surveillance that sequences at least ten percent of cases with a turnaround under two weeks should monitor cross-border spread and variant emergence through regional platforms [5,49]. Wildlife surveillance and occupational studies among hunters and bushmeat handlers should assess spillover risk [19-21]. Prospective evaluations of contact tracing, isolation, and vaccination should quantify real-world effectiveness in low-resource settings [23,49]. Qualitative studies on stigma, isolation, and vaccine acceptance should inform community engagement [50]. These priorities arise from gaps identified during rapid early characterization and support an evidence-based, scalable response.

## Conclusion

Rapid analysis of the first 44 mpox cases generated intelligence that continues to shape the outbreak response. Key findings –community-based transmission (95.5% no travel), household secondary attack rate 7.3% to 8.2%, two co-circulating sub-lineages, and exploratory risk factors – directly informed surveillance strategy shifts, genomic partnerships, and vaccine prioritization despite small sample size and statistical uncertainty. Translation of findings into action demonstrates practical utility of rapid characterization. Predominance of cases without travel informed the late March 2025 strategic shift from border-focused to community-based surveillance. Viral diversity established rationale for genomic surveillance partnerships achieving over 10% sequencing coverage (540 sequences) by October 2025. Exploratory risk factor analysis contributed to vaccine prioritization targeting healthcare workers, contacts, people living with HIV, adults 35 years or older, and persons with comorbidities when limited doses became available in March 2025. Consistency between initial patterns and those observed as the outbreak evolved to 5,426 confirmed cases with 58 deaths (case fatality rate 1.0%) across all 16 districts by October 2025 validates the relevance of early rapid characterization.

This work demonstrates both the value and challenges of outbreak response in resource-limited settings. Sierra Leone's response achieved substantial surveillance scale-up with 22,396 contacts traced, maintained genomic surveillance exceeding 10% sampling targets, and administered 170,141 vaccine doses by October 2025. However, vaccine coverage remained below 2.5% of the population due to dependence on donation channels rather than commercial procurement, exemplifying persistent global inequities in outbreak response capacity. Addressing these inequities requires sustained investment in African laboratory and surveillance capacity, equitable vaccine access mechanisms, and recognition that infectious disease threats anywhere threaten populations everywhere. Most importantly, this study illustrates that delaying outbreak analysis for statistical certainty may sacrifice the opportunity to guide early intervention when it matters most. Rapid analysis of early cases, despite small numbers and inherent uncertainties, provides actionable intelligence for guiding urgent decisions during the period when intervention can most effectively alter outbreak trajectories. The frameworks established through this early analysis-genomic surveillance partnerships, risk-based vaccine prioritization, and community-based case finding-scaled successfully as the outbreak grew, validating the approach. Future outbreak responses should embrace rapid early characterization while explicitly acknowledging uncertainty and planning for continuous validation as more data accumulate. The alternative-perfect analysis conducted too late-serves science better than public health.

### What is known about this topic

- Mpox Clade IIb emerged in 2022 with sustained human-to-human transmission, predominantly spreading through sexual networks in Europe and North America with genital lesions in 36-73% of cases;
- West and Central African mpox outbreaks exhibit primarily household and community-based transmission patterns with higher severity rates than observed in high-income countries;
- Africa remains underrepresented in mpox genomic surveillance despite bearing the highest historical disease burden, limiting real-time monitoring of viral evolution.

### What this study adds

- First integrated epidemiological and genomic characterization of Sierra Leone's 2025 mpox outbreak, documenting community-based transmission (95.5% without travel history) and household secondary attack rate of 7.3-8.2%;
- Identification of two co-circulating sub-lineages (A.2.2 and B.1.6) with strong APOBEC3 mutational signature, suggesting separate introductions from Nigeria and Côte d'Ivoire;
- Real-time risk stratification during the outbreak's initial phase that directly informed vaccine prioritization and surveillance strategy shifts, demonstrating the value of rapid early characterization.
